# Tissue plasminogen activator associated pulmonary hemorrhage in patient with *ACTA2*-associated arteriopathy

**DOI:** 10.1016/j.jvscit.2024.101558

**Published:** 2024-07-27

**Authors:** Travis G. Hughes, Mark A. Wurth, Mary B. Sheppard

**Affiliations:** a Division of Vascular and Endovascular Surgery, University of Kentucky, Lexington, KY; b Division of Pulmonology, Department of Pediatrics, College of Medicine, University of Kentucky, Lexington, KY; c Saha Cardiovascular Research Center, University of Kentucky, Lexington, KY; d Department of Family Medicine, University of Kentucky, Lexington, KY; e Department of Surgery, University of Kentucky, Lexington, KY

**Keywords:** ACTA2, Pulmonary hemorrhage, Brachial artery aneurysm, Thrombosis

## Abstract

Pulmonary hemorrhage after administration of tissue plasminogen activator (tPA) is a rare complication. We present a 15-year-old boy with a heterozygous pathogenic variant in *ACTA2* who presented with right brachial artery aneurysm thrombosis treated initially with tPA, resulting in pulmonary hemorrhage. We hypothesize that the underlying pulmonary abnormalities caused by the pathogenic *ACTA2* variant may be a risk factor for pulmonary hemorrhage after tPA administration.

Life-threatening hemorrhage secondary to thrombolysis has been observed to be as high as 5.6%.^1^ The incidence of pulmonary hemorrhage after administration of tissue plasminogen activator (tPA) is reported to be 0.39%.^2^ Emphysema is the only reported risk factor for diffuse pulmonary hemorrhage after the administration of tPA.^2^ *ACTA2* encodes the intracellular smooth muscle protein α-actin, a component of thin filaments, which interacts cyclically with thick filaments to enable cellular contraction.^3^ Pathogenic variants in *ACTA2* have pleomorphic effects. Known pulmonary complications are diverse, but can include defects in lung development/alveolarization, obstructive lung disease, and pulmonary hypertension.^4^, ^5^, ^6^

## Case report

A 15-year-old boy with a history of multisystem smooth muscle cell dysfunction syndrome secondary to ACTA2 (R179H), resulting in ascending aortic aneurysm, abdominal aortic ectasia, left axillary artery ectasia, and right brachial artery aneurysm, presented to an outside hospital with acute right upper extremity ischemia. At the time of presentation to outside hospital, he was 5′2″ and 47 kg. Coagulation studies revealed a hemoglobin/hematocrit of 15/42, platelet count of 263, international normalized ratio of 1.4, and an activated partial thromboplastin time of >200; he had received a bolus of heparin before transportation to the outside hospital. He was found to have a right brachial artery aneurysm thrombosis, resulting in Rutherford 2a ischemia. He underwent catheter-directed thrombolysis of the thrombosed right brachial artery aneurysm at a rate of 1 mg/h for 40 hours. During the lysis initiation and cessation, the patient underwent endotracheal intubation with general anesthesia; there is no documented injury to the oropharynx or airway with either procedure. The intention at the time of lysis initiation was to perform bypass and aneurysm ligation once the thrombus was cleared. Approximately 42 hours after cessation of tPA, he developed significant hemoptysis with a chest radiograph showing new multifocal opacities, supportive of a diagnosis of diffuse alveolar hemorrhage. At the time of his pulmonary hemorrhage his hemoglobin/hematocrit was 11/31, platelet count of 173, anti Xa of 0.3, international normalized ratio of 1.5, activated partial thromboplastin time of 137.4, and fibrinogen of 454. The patient developed respiratory failure requiring intubation. He underwent pulmonary angiography that did not show an arteriovenous fistula or nidus of bleeding. Bronchoscopy was unable to identify a focal source of bleeding. Rheumatologic workup revealed a positive anticardiolipin IgG and no other identified cause of autoimmune pulmonary vasculitis; he was treated empirically with steroids for possible antiphospholipid syndrome. Additionally, his postoperative course was complicated by frontotemporal seizure, acute kidney injury, and methicillin-resistant *Staphylococcus aureus* pneumonia; however, this complication was likely due to respiratory failure and critical illness. Before this event, he had no previous pulmonary disease or seizure. He ultimately required intubation for 40 days total. After his pulmonary hemorrhage, heparin was stopped; however, he was eventually transitioned to a low-dose heparin drip with a goal anti-Xa level of 0.3.

**Fig 1:**
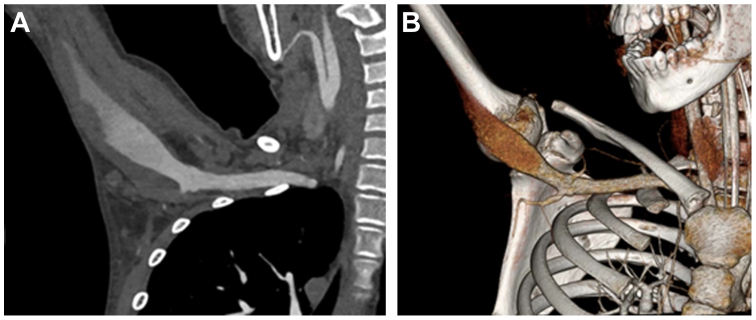
**(A)** Preoperative computed tomography angiography. **(B)** Preoperative reconstruction.

He was transferred to our facility because this was closer to home for his family. He remained intubated and had no signs of right upper extremity ischemia. Repeat computed tomography angiography showed persistent mural thrombus within the aneurysm. Multidisciplinary discussion concluded that patient would benefit from autologous axillary to brachial bypass and ligation of his aneurysm (Fig 1, *A* and *B*).

**Fig 2:**
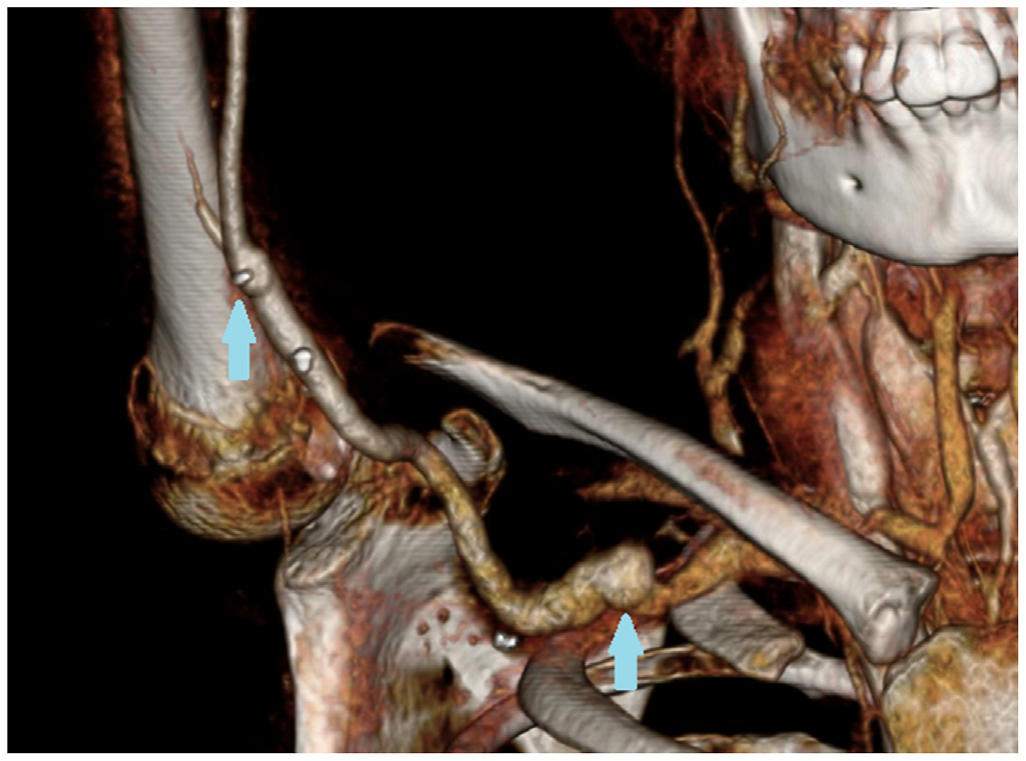
Postoperative reconstruction.

He was taken to the operating room and underwent right axillary to brachial bypass with saphenous vein bypass and ligation of a brachial artery aneurysm. The right saphenous vein was first harvested through two skip incisions. The brachial artery was dissected via a right longitudinal incision along the biceps groove. After proximal and distal control of the aneurysm was obtained, we proceeded with exposure of the axillary artery via an infraclavicular incision. We ligated the aneurysm, and then performed an axillary to brachial artery bypass with the harvested saphenous vein. After the bypass was performed, the aneurysm was opened and decompressed. All branches of the aneurysm were ligated. The patient was extubated on his first postoperative day. Two weeks later, he was discharged to inpatient rehabilitation on therapeutic enoxaparin sodium (Lovenox). He experienced no postoperative bleeding complications while therapeutically anticoagulated (Fig 2).

## Discussion

Actin alpha 2 (ACTA2) is an actin protein encoded by the *ACTA2* gene on chromosome 10. It is expressed in the smooth muscles of the aorta, peripheral arteries, central and peripheral veins, and urogenital system. Mutations in this gene are associated with smooth muscle dysfunction syndrome, which is characterized by a constellation of symptoms including congenital mydriasis, patent ductus arteriosus, pulmonary artery hypertension, arterial aneurysms, Moyamoya disease, intestinal hypoperistalsis, intestinal malrotation, and bladder hypotonia. Nearly every patient studied with this smooth muscle dysfunction syndrome has a patent ductus arteriosus or aortopulmonary window, and 36% of patients require elective aortic aneurysm repair at a median age of 14 years. Fifty-four percent of the studied patients have a peripheral arterial aneurysm; all of these aneurysms were in the carotid, brachiocephalic, subclavian, and axillary arteries. Axillary artery thrombosis was the most common complication of peripheral artery thrombosis. Pulmonary artery hypertension was observed in 48% of patients and chronic lung disease was observed in 33% of patients.^6^ We propose that this patient's underlying pulmonary disease placed him at higher risk for diffuse pulmonary hemorrhage in the setting of tPA administration. The mechanism could be related to a direct interaction between ACTA2 and tPA/PAI1 or it could be due to an indirect mechanism. Currently, it is not clear whether ACTA2 has any direct interactions with TPA or PAI1. We found a single article suggesting that interactions between PAI-1 and alpha-smooth muscle actin affect extracellular matrix remodeling in the setting of valve leaflet aneurysm formation, though their role in the larger vasculature remains uncertain.^7^ In terms of an indirect mechanism, emphysema is a known risk factor for diffuse pulmonary hemorrhage after the administration of tPA.^2^ Patients with ACTA2 variants are known to have significant structural lung abnormalities, including hyperinflation (suggestive of obstructive lung pathology), as well as lung cyst formation.^4^ This lung pathology may be sufficiently analogous to emphysema to account for the increased risk of pulmonary hemorrhage with administration of tPA. It is also possible that people with ACTA2 variants have an increased risk of pulmonary hemorrhage at baseline, as seen in people with autoimmune vasculitis and Goodpasture syndrome. However, this increased risk at baseline has not been documented in the literature to date and the patient's evaluation for vasculitis was negative. Pulmonary hemorrhage can also occur as a result of drugs/toxins, coagulopathy, mitral stenosis, and pulmonary veno-occlusive disease, but the patient's history and tests for these were negative.

## Conclusions

We report a case in which a 15-year-old boy with heterozygous pathogenic variant in *ACTA2* (R179H) developed acute right upper extremity ischemia secondary to a thrombosed right brachial artery aneurysm. His course was complicated by diffuse pulmonary hemorrhage after the initiation of thrombolysis. After recovery from his pulmonary hemorrhage, he underwent successful autologous bypass and ligation of his aneurysm. Diffuse alveolar hemorrhage is rare, but reported, complication of thrombolytic therapy. The only clearly identified risk factor is emphysema, which suggests that structural lung disease increases the risk of hemorrhage. We present a case of diffuse alveolar hemorrhage after tPA delivery in a patient with a heterozygous pathogenic variant in *ACTA2*. *ACTA2* variants have an association with chronic pulmonary disease, and affected individuals may be at increased risk for diffuse alveolar hemorrhage with thrombolytic therapy. A similar risk may apply to individuals with other heritable arteriopathy conditions that are also associated with underlying structural lung disease. Given the rarity of these cases, a full assessment of risk may be difficult to define and should generate further research interest moving forward.

## Disclosures

None.
